# Returning to Marathons: Complete Restoration of Exercise with Runner's Dystonia After Globus Pallidus Internus Deep Brain Stimulation

**DOI:** 10.1002/mds.70327

**Published:** 2026-04-29

**Authors:** Arthur Thevathasan, Kristian Bulluss, Katya Kotschet, Wesley Thevathasan

**Affiliations:** ^1^ Florey Department of Neuroscience and Mental Health The University of Melbourne Parkville Victoria Australia; ^2^ Department of Neurology Austin Health Heidelberg Victoria Australia; ^3^ Department of Neurosurgery Austin Health Heidelberg Victoria Australia; ^4^ Department of Neurosurgery St Vincent's Hospital Fitzroy Victoria Australia; ^5^ Department of Neurology St Vincent's Hospital Fitzroy Victoria Australia

**Keywords:** deep brain stimulation, dystonia, focal dystonia, globus pallidus, neuromodulation, programming

Runner's dystonia is a rare, adult‐onset, focal, task‐specific lower‐limb dystonia. Most cases have been described as treatment refractory. In contrast, we report a patient with runner's dystonia who sustained complete restoration of exercise function after contralateral globus pallidus internus deep brain stimulation (GPi‐DBS).

A 55‐year‐old right‐handed female elite‐level runner (~100 km/wk, formerly a national‐level competitor in Australia) developed left‐leg dystonia gradually over 5 years. Initially this was task specific, occurred only during running, but later also affected walking (to a lesser degree). The dystonia progressed from intermittent foot “slapping” to dystonic flexion of all toes and equinovarus foot posturing. Examination also revealed a suppressible, high knee lift, which we considered compensatory to avoid the foot catching rather than as a primary manifestation of dystonia. Burke‐Fahn‐Marsden Dystonia Rating Scale (BFMDRS) was 6. There was no dystonia at rest, geste antagoniste, relevant family history, or neuroleptic exposure. She exhausted therapeutic options, including physical therapy, trihexyphenidyl, levodopa, and botulinum toxin. A vesicular monoamine transporter dopamine scan was normal, with the patient declining neurogenetics testing.

Due to a substantial impact on quality of life, including inability to even walk her dog in a nearby park (walking tolerance <100 m), she underwent contralateral GPi‐DBS. Surgery was performed under general anesthesia using a stereotactic frame without complications. The target was the posteroventral GPi and adjacent subpallidal white matter, a region associated with optimal DBS outcomes for dystonia.^1^ The lead terminated just above the lateral aspect of the optic tract (20.5 mm lateral, 2.58 mm anterior, 3.88 mm inferior relative to the mid‐commissural point) (Fig. 1).

**FIG. 1:**
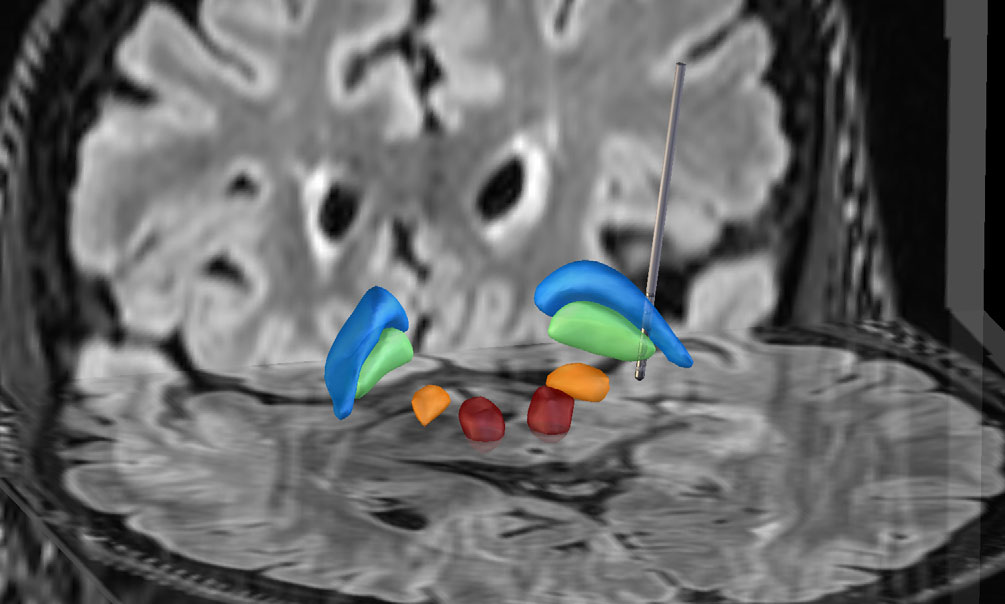
Reconstructed 3D images of the DBS (deep brain stimulation) lead in native space. Stimulation was delivered in equal fractionation to the distal two tiers, positioned predominantly in the subpallidal white matter, with the stimulation field likely to engage the GPi (globus pallidus internus) itself and pallidal output pathways. This is consistent with where DBS is reported to achieve optimal outcomes for dystonia.^1^ Red: red nucleus, orange: STN, green: GPi, blue: GPe. Images reconstructed using *Lead‐DBS*, version *3.2* (lead‐dbs.org). [Color figure can be viewed at wileyonlinelibrary.com]

Stimulation was fractionated equally between the bottom two tiers of the electrode in monopolar and gradually escalated to 6.9 mA, 60 μs, and 104 Hz at 12 weeks. She returned to running 6 km a day; however, she still experienced intermittent, minor symptoms. Stimulation was further escalated to 7.8 mA, which required a reduction in frequency to 85 Hz to escape a ceiling due to capsular side effects, a programming strategy described previously.^2^ Seven months post‐surgery, she ran the Melbourne marathon (in 3 hours 46 minutes) without symptoms. By this time, the patient considered herself asymptomatic. Objectively, at 1 year the BFMDRS was 1—with the only observable deficit being slight, barely evident foot posturing during walking and no deficits at all when running (Video 1).

**Video 1:** Pre‐DBS: (1) on walking in her home environment, the video demonstrates equinovarus posturing of the foot. Note the high knee lift was suppressible and not considered overflow from the dystonia. The patient was unable to run at this stage, with a video unable to be captured due to safety and risk of falling. (2) On sitting in the clinic, the video demonstrates action‐induced dystonic posturing on attempted plantar and dorsiflexion. One year post‐DBS: (1) on walking in her home environment, there is subtle dystonia still present, despite patient being asymptomatic. (2) Running is now possible with no dystonia obviously visible.

Although the GPi (and subpallidal white matter) is the conventional target for generalized and cervical dystonia, there is far less evidence for focal limb dystonia. GPi‐DBS has previously been reported to achieve a partial benefit for runner's dystonia^3^ and several cases of isolated task‐specific upper‐limb dystonia.^4^ Lesioning of a different target, the ventral‐oralis nucleus of the thalamus, has been successfully employed for focal limb dystonia, including complete resolution of runner's dystonia.^5^, ^6^

This report highlights that runner's dystonia can be a treatable condition and that the GPi is a reasonable DBS target, which in our patient proved capable of normalizing function—with a well‐placed electrode and sufficient current delivery.

## Author Roles

A.T.: direct care of patient, design, execution, analysis, writing, editing of the final version of the manuscript. K.B.: direct care of patient, execution, editing of the final version of the manuscript. K.K.: direct care of patient, execution, editing of the final version of the manuscript. W.T.: direct care of patient, design, execution, writing, editing of the final version of the manuscript.

## Full financial disclosures of all authors for the previous 12 months

A.T. is supported by the National Health and Medical Research Council (NHMRC) postgraduate research scholarship and the Australian and New Zealand Association of Neurologists (ANZAN). K.B., K.K., and W.T. have no other funding sources for the preceding 12 months.

## Data Availability

The data that support the findings of this study are available on request from the corresponding author. The data are not publicly available due to privacy or ethical restrictions.

## References

[1] Reich MM , Horn A , Lange F , et al. Probabilistic mapping of the antidystonic effect of pallidal neurostimulation: a multicentre imaging study. Brain 2019;142(5):1386–1398. 10.1093/brain/awz046 30851091

[2] Magown P , Andrade RA , Soroceanu A , Kiss ZHT . Deep brain stimulation parameters for dystonia: a systematic review. Parkinsonism Relat Disord 2018;54:9–16. 10.1016/j.parkreldis.2018.04.017 29705556

[3] Saminejad B , House P , Ballard DJ , Schrock L . Runner's dystonia successfully treated with unilateral Globus pallidus Interna (GPi) deep brain stimulation (DBS) (P1.043). Neurology 2016;86(16_supplement):P1.043. 10.1212/WNL.86.16_supplement.P1.043

[4] Doshi PK , Ramdasi RV , Karkera B , Kadlas DB . Surgical interventions for task‐specific dystonia (Writer's dystonia). Ann Indian Acad Neurol 2017;20(3):324. 10.4103/aian.AIAN_15_17 28904473 PMC5586136

[5] Horisawa S , Kohara K , Kawamata T , Taira T . Successful treatment of task‐specific lower extremity dystonia by ventro‐oral thalamotomy. Mov Disord 2018;33(2):338–339. 10.1002/mds.27180 29239020

[6] Horisawa S , Ochiai T , Goto S , et al. Safety and long‐term efficacy of ventro‐oral thalamotomy for focal hand dystonia. Neurology 2019;92(4):e371–e377. 10.1212/WNL.0000000000006818 30587520 PMC6345121

